# Thermodynamic models and factual data banks - ideal tools for the development of chemical processes

**DOI:** 10.1186/1758-2946-3-S1-O15

**Published:** 2011-04-19

**Authors:** Jürgen Gmehling

**Affiliations:** 1 University of Oldenburg, Technical Chemistry, 26111 Oldenburg, Germany

## 

The synthesis, design and simulation of chemical processes, in particular thermal separation processes is today carried out by solving the resulting balance equations of a mathematical model of the considered unit operation or the whole chemical plant using sophisticated commercial process simulation software.

The reliability and correctness of the simulation results is mainly influenced by the reliability and correctness of the thermophysical property parameters used for the pure compounds and their mixtures. For the description of the required phase equilibria G^E^-models and equations of state were developed. These models allow the prediction of the phase equilibrium behavior of multicomponent systems using binary experimental data only.

However, since the number of experimental binary data is limited, these methods often cannot be applied. Therefore in particular for process development reliable predictive models with a large range of applicability are most important.

With the help of the worldwide largest factual data bank (Dortmund Data Bank (DDB)) for pure component properties, phase equilibria and excess properties, in the last 35 years powerful group contribution methods (UNIFAC, modified UNIFAC) have been developed in my research group. By combination of cubic equations of state with the group contribution concept, now even the phase equilibrium behavior with supercritical compounds can be predicted. At the same time other important properties (e.g. densities, enthalpies, heat capacities, etc.) can directly be predicted. With the development of an adequate electrolyte model (LIQUAC, LIFAC) the equation of state approach was even extended to systems with strong electrolytes. Today ideal predictive thermodynamic tools are available, which can be used in combination with factual data banks for the development of chemical processes.

In the lecture the status of the factual data bank and the different predictive models will be shown. Furthermore important applications of industrial interest of the predictive thermodynamic models and the Dortmund Data Bank will be presented.

